# Awareness of Pregnant Patients about Congenital Cytomegalovirus Infection—A Semi-Systematic Review

**DOI:** 10.3390/jcm13092586

**Published:** 2024-04-28

**Authors:** Paweł Bartnik, Aleksandra Bender, Joanna Kacperczyk-Bartnik, Michał Ciebiera, Aleksandra Urban, Anna Sienko, Esra Bilir, Ewa Romejko-Wolniewicz, Jacek Sieńko

**Affiliations:** 1 II Department of Obstetrics and Gynaecology, Medical University of Warsaw, 02-091 Warsaw, Polandaleksandra.urban@wum.edu.pl (A.U.); ewa.romejko-wolniewicz@wum.edu.pl (E.R.-W.); jacek.sienko@wum.edu.pl (J.S.); 2 Students’ Scientific Group, II Department of Obstetrics and Gynaecology, Medical University of Warsaw, 02-091 Warsaw, Poland; 3 Second Department of Obstetrics and Gynecology, Centre of Postgraduate Medical Education, 00-189 Warsaw, Poland; 4 Warsaw Institute of Women’s Health, 00-189 Warsaw, Poland; 5 School of Clinical Medicine, University of Cambridge, Cambridge CB2 0SP, UK; 6 Department of Global Health, Koç University Graduate School of Health Sciences, İstanbul 34450, Turkey; 7 Department of Obstetrics and Gynecology, University Hospitals Schleswig-Holstein, Campus Kiel, 24105 Kiel, Germany

**Keywords:** awareness, congenital, cytomegalovirus infections, pregnancy

## Abstract

**Background**: Cytomegalovirus (CMV) infection represents a major issue worldwide, since it constitutes the most common viral congenital infection, with a prevalence of 0.58% and 1–5% in developed and developing countries, respectively. According to recent studies, prenatal treatment significantly decreases the risk of vertical CMV transmission, and early intervention may even prevent the termination of pregnancy. This study aimed to investigate the level of awareness of CMV among pregnant patients through a semi-systematic review. **Methods**: We included all of the original articles investigating knowledge and awareness about CMV infection among pregnant women. Our research included the PubMed database. Following the Preferred Reporting Items for Systematic Reviews and Meta-Analyses (PRISMA) 2020 statement, the Covidence system automatically guided us to screen the titles and/or abstracts, and then full-texts, followed by data extraction from the eligible studies. **Results**: We screened 764 studies altogether, with 13 studies included in this analysis. Knowledge about the existence of CMV infection risk varied between the articles, ranging from 11.4% in a study performed in Ireland to 60% reported in a study on the French population. Studies analyzing the impact of educational interventions on patients’ knowledge about preventive measures reported significant improvement compared to their level of awareness before the intervention. **Conclusions**: Patients’ awareness and knowledge about CMV seemed to be generally low or very low during the last decade before the development of effective secondary prevention methods. Educational interventions seem to be effective, and therefore their wide use could be of potential benefit. In the era of available secondary prevention of vertical transmission, it is crucial to concentrate the efforts of different stakeholders to increase the awareness of cCMV among pregnant women.

## 1. Introduction

Cytomegalovirus (CMV) is a DNA virus belonging to the *Herpesviridae* family [1]. Prenatal CMV infection represents a major issue worldwide since it constitutes the most common viral congenital infection, with a prevalence of 0.58% and 1–5% in developed and developing countries, respectively [2,3,4,5]. The concern is primarily associated with the risk of long-term complications for the affected individuals. Notably, congenital neurological defects, particularly non-genetic sensorineural hearing loss (SNHL), are predominantly caused by CMV, both in cases of primary and non-primary maternal infection [6]. SNHL is observed in approximately 10.7–32.4% of all children diagnosed with congenital CMV (cCMV) [7]. Other complications include several ophthalmologic manifestations, developmental delays, and neuroimaging abnormalities [8,9,10]. Furthermore, these possible sequelae necessitate prolonged medical observation and various therapeutic interventions. This has prompted further research and the revision of screening and treatment protocols.

Regarding the possibilities of secondary prevention and prenatal treatment, many limitations were concerned with the potential teratogenic and mutagenic effects of valganciclovir [11]. According to recent studies, valacyclovir treatment significantly decreases the risk of vertical CMV transmission, and early intervention may even prevent the termination of pregnancy [12,13,14]. Valacyclovir is characterized by pregnancy category B, according to the Food and Drug Administration (FDA) [15]. With concentrations ten and seven times higher than in human plasma levels, no teratogenic effect in rodents was observed [15]. Therefore, valacyclovir treatment is recognized for the secondary prevention of cCMV. However, it is important to emphasize that the basis for preventing cCMV infection remains primary prevention among pregnant women. This includes compliance with principles of hygiene such as washing hands after changing diapers or touching toys, avoiding contact with contaminated bodily fluids, including saliva, urine, breast milk, genital secretions, and plasma, and refraining from sharing food or drinks with young children or kissing them. Consequently, raising awareness about the risk of cCMV infection among patients might mitigate the issue by preventing primary infection and, if needed, initiating antiviral therapy during gestation. Nonetheless, this would only be possible with proper patient awareness.

This study aimed to investigate the level of the awareness of cCMV among the population of pregnant patients through a semi-systematic review. We investigated three different areas of knowledge about cCMV: the infection itself, preventive measures, and complications following infection.

## 2. Materials and Methods

### 2.1. Selection of Studies

We included all of the original articles investigating knowledge and awareness about cCMV infection among pregnant women. Additionally, our inclusion criteria comprised a requirement for the availability of the full text in the English language. We excluded studies enrolling non-pregnant individuals or postpartum women. We used Covidence software (Veritas Health Innovation Ltd., Melbourne, Australia) for study selection and data extraction [16]. Two co-authors (J.K.B. and A.U.) screened the studies for their eligibility. In cases of discrepancy, a third co-author (E.B. or P.B.) was consulted. Following the Preferred Reporting Items for Systematic Reviews and Meta-Analyses (PRISMA) 2020 statement, the Covidence system automatically guided us to screen first the titles and/or abstracts, and then perform full-text reviews, followed by data extraction from the eligible studies [16,17].

### 2.2. Search Strategy

Our research included the PubMed database. We defined the population as pregnant women, both primiparous and multiparous, as awareness during pregnancy is crucial for the primary and secondary prevention of cCMV. To address our research question, we used Medical Subject Headings (MeSH) of the relevant terms for cCMV and neonate, as well as various synonyms of ‘awareness’. Our PubMed advanced search query was the following: ((((((CMV) OR (Human cytomegalovirus)) OR (Cytomegalovirus)) OR (cytomegalovirus infection)) OR (HCMV)) AND ((((((awareness) OR (knowledge)) OR (attitude)) OR (Perception)) OR (Familiarity)) OR (Education))) AND ((((((congenital) OR (Inborn)) OR (neonatal)) OR (perinatal)) OR (neonate)) OR (fetal)). The search was performed on 30 January 2024. Our study did not employ any additional search strategies beyond the initial approach.

### 2.3. Data Extraction

Following the completion of the study selection process, we compiled the following information from the full text: authors’ names, publication year, country, sample size, data collection method, and the results of questions inquiring about general information about cCMV infection, primary preventive measures (PPM), and possible complications.

## 3. Results

**Figure 1:**
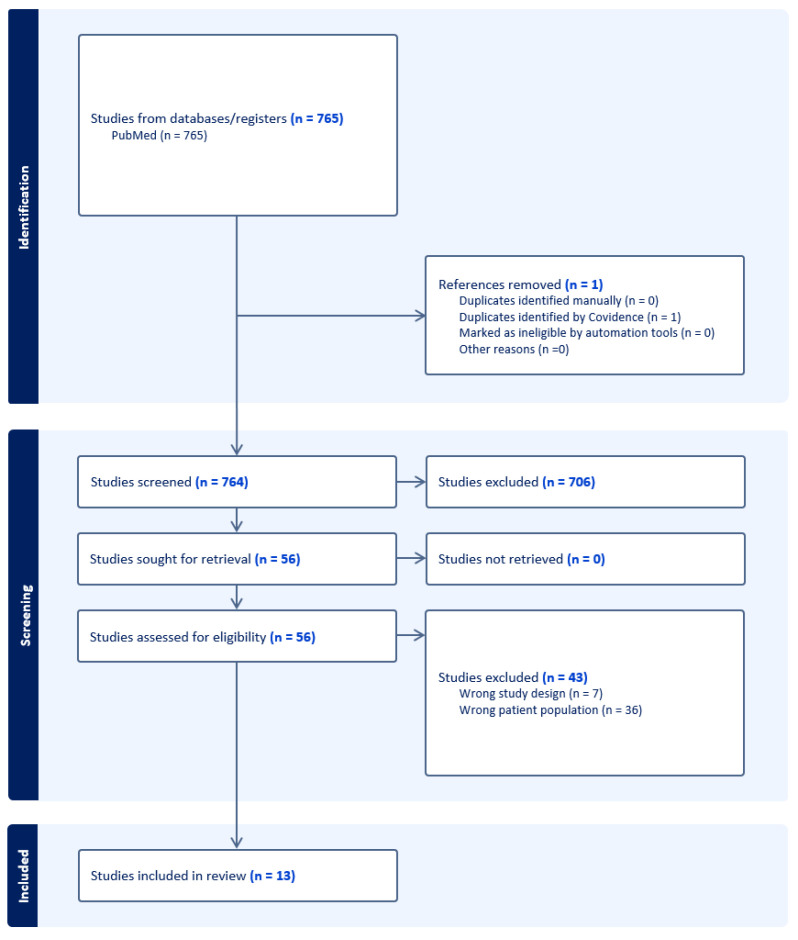
PRISMA flow diagram of the screening process.

We screened 764 studies altogether, including 13 studies in the final analysis (Figure 1). The detailed results of a review of the selected studies are presented in Summary Table 1. Knowledge about the existence of cCMV infection risk varied between the studies, ranging from 11.4% in a study by Basit et al. performed in Ireland to 60% reported in a study on the French population by Cordier et al. [18,19]. The baseline level of knowledge about the possible primary prevention of cCMV infection was rather unsatisfactory. Studies analyzing the impact of educational interventions on patients’ knowledge about preventive measures reported significant improvement compared to the levels of awareness before the intervention [20,21,22]. The most commonly identified complication of cCMV infection was hearing loss; however, in the majority of cases, the studied population initially had little to no knowledge of complications associated with CMV infection in pregnancy.

## 4. Discussion

In the results of our review, the complex issue of cCMV awareness among pregnant women emerges as a multifaceted concern warranting a comprehensive examination. Numerous insightful observations can be extracted from our analysis.

Based on the studies included in our review, it is noteworthy that all analyzed studies, even if published after 2020, described the awareness of populations evaluated predating this year (Table 1). It is significant in the light of the publication of the first randomized controlled trial (RCT) proving the effectiveness of high doses of oral valacyclovir in the secondary prevention of cCMV [12]. The very study mentioned has changed the general approach toward cCMV, especially following a recent meta-analysis from 2024, which included two additional observational studies confirming the effectiveness of valacyclovir [31]. Therefore, the lack of proven and effective secondary prophylaxis at the time of all of the analyzed awareness studies should be taken into account.

The general awareness about cCMV in all of the analyzed studies was considered to be low or very low—the lowest percentage of patients who declared an awareness of cCMV was 11.4% in the Irish population (Table 1) [18]. It is important to notice that all of the analyzed study groups were recruited from developed countries (Table 1). In 11 out of 13 analyzed articles, the declared awareness ratio did not exceed 40% of the studied groups [20,21,22,23,24,25,27,28,29,30]. The two exceptions were the Italian study by Vena et al., which reported a ratio of 59.1%, and the French study by Cordier et al., which showed a general ratio of knowledge of 60% [18,24]. Even those relatively satisfactory awareness ratios should be approached with caution. When patients in the study by Vena et al. were further asked about primary preventive measures against cCMV infection, only 8.4% of respondents correctly identified all three proposed behaviors [26]. Based on these findings, it can be suspected that respondents could overestimate their knowledge and understanding of what cCMV is. Furthermore, in the study by Cordier et al., the analyzed group consisted of patients from a university hospital, in which 74% of respondents declared CMV awareness, and from a general hospital, where only 34% of respondents stated the same [19]. The ratio collected in the latter hospital is similar to other studies and can be considered to be more representative of the general population.

As the general knowledge of cCMV was low, one-third of the analyzed studies tried to identify demographical factors associated with higher levels of cCMV awareness [18,19,27]. Higher cCMV awareness was associated with specific demographic factors traditionally typical for higher socioeconomic status: higher education [18,19], non-migrant status [18], and employment status, especially in the healthcare sector [27]. Unfortunately, no study examined the association between demographic factors and not only CMV awareness, but also the knowledge of primary preventive methods or actual preventive behaviors.

Only one of the analyzed studies, performed by Suga et al., tried to estimate the changes in cCMV awareness over time [30]. The study period was 6 years and was divided into four periods; no significant changes were observed. No specific educational intervention was performed over the examined period. Similarly, upon comparing the overlooked findings of all of the studies, no consistent trend toward changes in CMV awareness can be seen; the study with the lowest knowledge ratio was from 2019, while the one with the highest was from 2012 [18,19]. The studies were of heterogeneous design and explored different patient groups.

The knowledge about effective PPM in the analyzed studies is, in most cases, considered even poorer than the general awareness of cCMV. Among the included studies, nine analyzed patients’ knowledge about PPM, one investigated the use of PPM, and one reported patients’ attitudes towards PPM. Across all studies (six in total), in which comparison was feasible, the percentage of correct answers regarding various PPM was lower than the declared knowledge or awareness about cCMV [18,19,25,26,28,30]. The study that analyzed attitudes towards the proposed PPM found a generally positive outlook, with a strong belief in their overall ease of application [27]. The study that analyzed the use of PPM focused on the effect of educational intervention and did not provide specific ratios of initial PPM use [20].

The knowledge about the complications of cCMV was analyzed only in six out of thirteen of the included studies [19,20,21,22,25,29]. One of the studies, unfortunately, did not present its results on this topic at all [21]. The general knowledge about the complications of cCMV did not differ from the previously analyzed two topics, and could be considered to be low or very low. Knowledge about severe complications of cCMV did not exceed 20% of the included patients, except in the aforementioned French study by Cordier et al., in which the neurological complications following cCMV were correctly identified by up to 53% [19]. In this case, however, the population in the study was highly specific and consisted of a large proportion of women from a university hospital.

An interesting topic studied by three of the analyzed studies was the effectiveness of educational interventions [20,21,23]. The authors of the studies agreed that, in the context of low knowledge about cCMV in the general population, there is an urgent need to change that situation. All of the studies proved the significant positive impact of educational interventions, which ranged from educational videos to educational leaflets. The positive impact was seen in all of the analyzed aspects—knowledge about cCMV, PPM, and the complications. The English study by Colbert et al. was designed as a pilot study for a wider intervention in the general UK population, and this context proved the possible effectiveness of educational interventions for the general population [20]. All of the mentioned studies, unfortunately, lacked an analysis of the effectiveness on actual cCMV infection rates and cost-effectiveness calculations.

Considering the recent findings on effective secondary prevention and prenatal treatment opportunities, it is even more clinically relevant to provide pregnant patients with access to reliable educational resources and increase their awareness about cCMV [12,13,31]. One of the strengths of this semi-systematic review is understanding the general perspective and current knowledge status, as well as the identification of areas for improvement due to the inspection of studies including pregnant patients. We decided to include all pregnant patients as they are the target group for secondary prevention during pregnancy. Without their knowledge about the existence and consequences of cCMV, it is not possible to effectively introduce prenatal treatment and avoid/decrease the severity of vertical infections. Our study also indicated a lack of accessible data on the awareness of pregnant women from the low- and middle-income countries and identified a need for adequate research in those settings.

In the opinion of the authors of the review, future studies analyzing CMV awareness and various educational interventions should include knowledge about effective secondary prevention in the form of high doses of valacyclovir. The presented results support a high demand for updating societies’ guidelines to include the possibility of secondary prevention and prenatal treatment for cCMV as well as proposals for increasing pregnant patients’ awareness of cCMV [12,31,32].

## 5. Conclusions

Patients’ awareness and knowledge about cCMV in developed countries seemed to be generally low or very low during the last decade before the development of effective secondary prevention methods. Educational interventions seem to be effective, and therefore their wide use could be of potential benefit. In the era of available secondary prevention of vertical transmission, it is crucial to concentrate the efforts of different stakeholders to increase the awareness of cCMV among pregnant women.

## Figures and Tables

**Table 1:** Data extraction results.

	Authors	Publication Year	Country	Study Population	Data Collection Method	Assessed Areas of Knowledge		
						Infection	Preventive Measures	Complications
1.	Schaefer et al. [21]	2020	USA	263 pregnant women < 34 weeks of gestation	Outpatient clinic, two self-reported questionnaires—initial and 2–4 weeks post-education	A total of 33% of patients declared knowledge about cCMV prior to intervention; this increased to 75% after educational intervention (*p* < 0.001).	PPM was well-accepted by the patients in the initial questionnaire; after educational intervention significant improvement in washing hands after handling toys (*p* < 0.001); avoiding children’s saliva (*p* < 0.007); and washing hands after wiping children’s face (*p* < 0.01).	Present in the questionnaires, results not presented.
2.	Beaudoin et al. [23]	2021	Canada	234 pregnant women 11–16 weeks of gestation	Self-administered questionnaire at obstetrics outpatient unit after educational intervention	A total of 74.4% of patients were unaware of the risk of cCMV infection (before educational intervention).	Two questions about PPM based on the Likert scale. The median scores were 3.75/5 for avoiding sharing behaviors and 4.0/5 for not kissing a child on the lips.	Not analyzed.
3.	Greye et al. [24]	2022	Germany	1233 pregnant women	Interviewer or self-administered questionnaire in five hospital-based maternity units	A total of 38% of all patients were educated about cCMV—the lowest number in comparison to other congenital infections.	Not analyzed.	Not analyzed.
4.	Monteiro et al. [25]	2023	Portugal	80 pregnant women in the third trimester	Self-reported questionnaire in a single secondary center	Knowledge about cCMV was estimated to be low (<4/10 Y/N answers) in 56.3% of patients, medium in 38.8% of cases (>3/10 and <8/10 Y/N answers), and high in 5.0% (>7/10 Y/N answers) of the patients.	Three questions about PPM measures—correct answers ratio spanned from 23.8% to 27.5%.	Simple, single question about the possibility of birth defects associated with cCMV—60% of respondents answered correctly.
5.	Vena et al. [26]	2021	Italy	296 pregnant patients attending antenatal visits	Self-reported questionnaire	A total of 59.1% of patients declared knowledge about cCMV.	A total of 25% of patients recognized at least one correct PPM, 11% recognized two, and 8.4% correctly recognized all proposed PPM.	Not analyzed.
6.	Calvert et al. [20]	2021	UK	103 pregnant patients attending antenatal visits living with at least one child younger than 4 years old	Self-administered questionnaire at baseline and after randomized educational intervention at 34 weeks of gestation	A total of 36% of respondents were “familiar” with cCMV at baseline; targeted educational intervention significantly improved general knowledge about cCMV.	Targeted educational intervention significantly improved knowledge about cCMV prevention.	Targeted educational intervention significantly improved knowledge about cCMV complications.
7.	Lim, Tan & Tan [27]	2012	Singapore	200 pregnant patients attending antenatal visits.	Self-administered questionnaire at the outpatient clinic.	A total of 20% of respondents were aware of cCMV, more commonly in the healthcare workers group (55%; *p* < 0.001). No other factors associated with cCMV awareness were found to be significant.	Knowledge about PPM not analyzed. Attitude towards CDC-proposed PPM was found to be positive—guidelines were found to be “easy” or “very easy” to implement by between 81.7% to 98.0% of respondents.	Not analyzed.
8.	Morioka et al. [28]	2014	Japan	342 pregnant women at various stages of pregnancy	Self-administered questionnaire completed at University Hospital.	A total of 18% of respondents were aware of cCMV, 8% correctly reported infection sources.	A total of 4% reported definite knowledge about PPM, 7% reported some knowledge, and 85% had no knowledge.	Not analyzed.
9.	Kobayashi et al. [29]	2021	Japan	535 pregnant women at various stages of pregnancy and 571 men and non-pregnant women from the general population.	A web-based self-reported questionnaire among selected patients.	A total of 16.1% of pregnant women were aware of cCMV (10.2% in the general population; *p* < 0.004);	Among the PPM proposed by the authors, “Wash hands after diaper changing” was correctly chosen by 43.0% of pregnant women, “Avoid kissing young children on the mouth” by 46.5%, “Do not share food, drink, or cutlery with young children” by 41.9%, and “Use a condom during sexual intercourse” by 19.8%. No differences from the general population were observed.	cCMV sequelae correctly chosen by the patients were hearing loss—22.1%, developmental delay—16.3%, motor delay—14.0%, epilepsy—5.8%, and visual problems—17.4%. No differences from the general population were observed.
10.	Suga et al. [30]	2021	Japan	1144 pregnant women	Self-administered questionnaire at University Hospital	A total of 33% of patients were aware of cCMV. Awareness of cCMV did not change significantly during the observation period (2012–2018).	Among patients aware of cCMV, 27.2% of patients knew PPM.	Not analyzed.
11.	Lazzaro et al. [22]	2019	Australia	457 pregnant women	Two self-administered questionnaires at two maternity hospitals in pregnancies < 32 weeks of gestation and after informational intervention.	16% of patients reported knowledge about cCMV existence.	A total of 55% initially vs. 99% after education identified thorough hand-washing as PPM (*p* < 0.001); 35% initially vs. 98% identified using the same cutlery as a child as effective PPM (*p* < 0.001); 36% vs. 98% identified not kissing a child on the mouth as PPM (*p* < 0.001); and 34% vs. 94% identified not touching a child’s urine or running nose correctly (*p* < 0.001).	Initially, 35% of women knew about deafness caused by c CMV—after educational intervention this was 85% (*p* < 0.001); initially, 38% knew about learning problems in children—in the second questionnaire this was 81% (*p* < 0.001).
12.	Basit et al. [18]	2019	Ireland	282 pregnant women	Self-administered questionnaire at the antenatal visit (12–14 weeks of gestation)	A total of 11.4% of women knew about cCMV. Higher awareness was significantly associated with first language not being English (*p* < 0.001), being born outside Ireland (*p* < 0.001), and increasing education levels. (*p* < 0.03).	Correct answers for PPM were as follows: “washing hands with soap and water after changing a child’s nappy”—19.5%, “not sharing food, drink or other utensils with younger children”—7.9%, “not kissing young children under the age of 5 or 6 years on the mouth and the cheek”—6.5%.	Not analyzed.
13.	Cordier et al. [19]	2012	France	362 pregnant women	Self-administered questionnaire at the antenatal visits in two hospitals—one university hospital with a policy of CMV education and a general hospital without such a policy.	A total of 60% of all patients knew of cCMV (74% in a university hospital vs. 34% in a general hospital; *p* < 0.001). Awareness was statistically associated with the hospital’s CMV information policy, higher maternal educational level, higher parity, and field of employment (healthcare).	A total of 92% of the patients recognized frequent hand washing as a PPM; 86% knew avoiding sharing cups, plates, utensils, and food is an effective PPM; 89% knew to avoid kissing on the mouth; and 72.4% of patients knew all of the presented hygiene rules.	Hearing loss and mental delay were identified as results of congenital CMV infection by 42% and 53% of all patients who knew about cCMV.

## Data Availability

All obtained datasets are included in this publication.

## References

[1] Francki R.I.B., Fauquet C.M., Knudson D.L., Brown F. (2012). Classification and Nomenclature of Viruses: Fifth Report of the International Committee on Taxonomy of Viruses. Virology Division of the International Union of Microbiological Societies.

[2] Goderis J., De Leenheer E., Smets K., Van Hoecke H., Keymeulen A., Dhooge I. (2014). Hearing loss and congenital CMV infection: A systematic review. Pediatrics.

[3] Manicklal S., Emery V.C., Lazzarotto T., Boppana S.B., Gupta R.K. (2013). The “silent” global burden of congenital cytomegalovirus. Clin. Microbiol. Rev..

[4] Ssentongo P., Hehnly C., Birungi P., Roach M.A., Spady J., Fronterre C., Wang M., Murray-Kolb L.E., Al-Shaar L., Chinchilli V.M. (2021). Congenital Cytomegalovirus Infection Burden and Epidemiologic Risk Factors in Countries with Universal Screening: A Systematic Review and Meta-analysis. JAMA Netw. Open.

[5] Rybak-Krzyszkowska M., Górecka J., Huras H., Massalska-Wolska M., Staśkiewicz M., Gach A., Kondracka A., Staniczek J., Górczewski W., Borowski D. (2023). Cytomegalovirus Infection in Pregnancy Prevention and Treatment Options: A Systematic Review and Meta-Analysis. Viruses.

[6] Maltezou P.G., Kourlaba G., Kourkouni Ε., Luck S., Blazquez-Gamero D., Ville Y., Lilleri D., Dimopoulou D., Karalexi M., Papaevangelou V. (2020). Maternal type of CMV infection and sequelae in infants with congenital CMV: Systematic review and meta-analysis. J. Clin. Virol..

[7] Fletcher K.T., Horrell E.M.W., Ayugi J., Irungu C., Muthoka M., Creel L.M., Lester C., Bush M.L. (2018). The Natural History and Rehabilitative Outcomes of Hearing Loss in Congenital Cytomegalovirus: A Systematic Review. Otol. Neurotol..

[8] Pesch M.H., Schleiss M.R. (2022). Emerging Concepts in Congenital Cytomegalovirus. Pediatrics.

[9] Zhou Y.P., Mei M.J., Wang X.Z., Huang S.N., Chen L., Zhang M., Li X.Y., Qin H.B., Dong X., Cheng S. (2022). A congenital CMV infection model for follow-up studies of neurodevelopmental disorders, neuroimaging abnormalities, and treatment. JCI Insight.

[10] Rybak-Krzyszkowska M., Górecka J., Huras H., Staśkiewicz M., Kondracka A., Staniczek J., Górczewski W., Borowski D., Grzesiak M., Krzeszowski W. (2023). Ultrasonographic Signs of Cytomegalovirus Infection in the Fetus-A Systematic Review of the Literature. Diagnostics.

[11] Pikis A. Clinical Review: Valcyte^®^ (Valganciclovir Hydrochloride). New Drug Application NDA 22-257/SN00 and sNDA 21-304/SN07. https://www.fda.gov/files/drugs/published/22257-Valganciclovir-Clinical-PREA.pdf.

[12] Shahar-Nissan K., Pardo J., Peled O., Krause I., Bilavsky E., Wiznitzer A., Hadar E., Amir J. (2020). Valaciclovir to prevent vertical transmission of cytomegalovirus after maternal primary infection during pregnancy: A randomised, double-blind, placebo-controlled trial. Lancet.

[13] D’Antonio F., Marinceu D., Prasad S., Khalil A. (2023). Effectiveness and safety of prenatal valacyclovir for congenital cytomegalovirus infection: Systematic review and meta-analysis. Ultrasound Obstet. Gynecol..

[14] Zammarchi L., Tomasoni L.R., Liuzzi G., Simonazzi G., Dionisi C., Mazzarelli L.L., Seidenari A., Maruotti G.M., Ornaghi S., Castelli F. (2023). Treatment with valacyclovir during pregnancy for prevention of congenital cytomegalovirus infection: A real-life multicenter Italian observational study. Am. J. Obstet. Gynecol. MFM.

[15] VALTREX^®^ (Valacyclovir Hydrochloride) Caplets Prescribing Information. New Drug Application NDA 20-487/S-007. https://www.accessdata.fda.gov/drugsatfda_docs/label/2010/021304s008,022257s003lbl.pdf.

[16] Covidence Systematic Review Software, Veritas Health Innovation, Melbourne, Australia. www.covidence.org.

[17] Page M.J., McKenzie J.E., Bossuyt P.M., Boutron I., Hoffmann T.C., Mulrow C.D., Shamseer L., Tetzlaff J.M., Akl E.A., Brennan S.E. (2021). The PRISMA 2020 statement: An updated guideline for reporting systematic reviews. Bmj.

[18] Basit I., Crowley D., Geary M., Kirkham C., Mc Dermott R., Cafferkey M., Sayers G. (2019). Awareness and Preventative Behaviours Regarding Toxoplasma, Listeria and Cytomegalovirus Among Pregnant Women. Ir. Med. J..

[19] Cordier A.G., Guitton S., Vauloup-Fellous C., Grangeot-Keros L., Ayoubi J.M., Benachi A., Picone O. (2012). Awareness of cytomegalovirus infection among pregnant women in France. J. Clin. Virol..

[20] Calvert A., Vandrevala T., Parsons R., Barber V., Book A., Book G., Carrington D., Greening V., Griffiths P., Hake D. (2021). Changing knowledge, attitudes and behaviours towards cytomegalovirus in pregnancy through film-based antenatal education: A feasibility randomised controlled trial of a digital educational intervention. BMC Pregnancy Childbirth.

[21] Schaefer M.R., Holttum J., Olson M., Westenberg D., Rubin N., Schleiss M.R., Nyholm J. (2020). Development and Assessment of a Prenatal Cytomegalovirus (CMV) Educational Survey: Implementation and Impact in a Metropolitan University-Based Clinic. Int. J. Womens Health.

[22] Lazzaro A., Vo M.L., Zeltzer J., Rawlinson W., Nassar N., Daly K., Lainchbury A., Shand A. (2019). Knowledge of congenital cytomegalovirus (CMV) in pregnant women in Australia is low, and improved with education. Aust. N. Z. J. Obstet. Gynaecol..

[23] Beaudoin M.L., Renaud C., Boucher M., Kakkar F., Gantt S., Boucoiran I. (2021). Perspectives of women on screening and prevention of CMV in pregnancy. Eur. J. Obstet. Gynecol. Reprod. Biol..

[24] Greye H., Henning S., Freese K., Köhn A., Lux A., Radusch A., Redlich A., Schleef D., Seeger S., Thäle V. (2022). Cross-sectional study to assess awareness of cytomegalovirus infection among pregnant women in Germany. BMC Pregnancy Childbirth.

[25] Monteiro S., Gonçalves A., Torrão M.M., Costa V., Almeida A. (2023). Knowledge of cytomegalovirus and available prevention strategies in pregnancy: A cross-sectional study in Portugal. J. Matern. Fetal Neonatal Med..

[26] Vena F., D’Ambrosio V., Pajno C., Boccherini C., Corno S., Di Mascio D., Piccioni M.G., Salerno M.G., Bisogni F., Brunelli R. (2021). Pregnant women’s knowledge and behaviour to prevent cytomegalovirus infection: An observational study. J. Perinat. Med..

[27] Lim S.L., Tan W.C., Tan L.K. (2012). Awareness of and attitudes toward congenital cytomegalovirus infection among pregnant women in Singapore. Int. J. Gynaecol. Obstet..

[28] Morioka I., Sonoyama A., Tairaku S., Ebina Y., Nagamata S., Morizane M., Tanimura K., Iijima K., Yamada H. (2014). Awareness of and knowledge about mother-to-child infections in Japanese pregnant women. Congenit. Anom..

[29] Kobayashi M., Okahashi A., Okuyama K., Hiraishi N., Morioka I. (2021). Awareness and knowledge of congenital cytomegalovirus infection among pregnant women and the general public: A web-based survey in Japan. Environ. Health Prev. Med..

[30] Suga S., Fujioka K., Nakasone R., Abe S., Fukushima S., Ashina M., Nishida K., Nozu K., Iijima K., Tanimura K. (2021). Changes in awareness and knowledge concerning mother-to-child infections among Japanese pregnant women between 2012 and 2018. PLoS ONE.

[31] Chatzakis C., Shahar-Nissan K., Faure-Bardon V., Picone O., Hadar E., Amir J., Egloff C., Vivanti A., Sotiriadis A., Leruez-Ville M. (2024). The effect of valacyclovir on secondary prevention of congenital cytomegalovirus infection, following primary maternal infection acquired periconceptionally or in the first trimester of pregnancy. An individual patient data meta-analysis. Am. J. Obstet. Gynecol..

[32] Coll O., Benoist G., Ville Y., Weisman L.E., Botet F., Maurizio M., Anceschi T.W.P.I.W.G., Greenough A., Gibbs R.S., Carbonell-Estrany X. (2009). Guidelines on CMV congenital infection. J. Perinat. Med..

